# Supplementary material for: Quantum control and characterization of ultrafast ionization with orthogonal two-color laser pulses
Source: Sci Rep. 2020 Jan 14;10:239. doi: 10.1038/s41598-019-57125-z (PMC6959349; doi:10.1038/s41598-019-57125-z)
Supplement: Supplementary file 1 — Supplementary Information. [file 41598_2019_57125_MOESM1_ESM.pdf]

Supplemental Material to "Quantum control and characterization of ultrafast ionization with orthogonal two-color laser pulses"

Hicham Agueny

In this supplementary material we provide additional calculations based on solving the time-dependent Schrodinger equation (TDSE) in three-dimension (3D) using a one-color scheme. This is done to support the 2D calculations performed in the main manuscript. Here the TDSE is written in cylindrical coordinates

$$\Big[H_0 + H_I(t) - i\frac{\partial}{\partial t}\Big]\Psi(\vec{r}, t) = 0. \tag{1}$$

where $\vec{r} = (\rho, z, \phi)$ denotes the vector position of the electron. The field-free Hamiltonian H_0 is transformed into

$$H_0 = -\frac{1}{2}\frac{\partial^2}{\partial z^2} + -\frac{1}{2}\frac{\partial^2}{\partial \rho^2} + \frac{(m^2 - \frac{1}{4})}{2\rho^2} - \frac{1}{\sqrt{\rho^2 + z^2}}. \tag{2}$$

by performing the mathematical transformation $\Psi(\vec{r}) = \psi(\rho, z)e^{im\phi}/\sqrt{2\pi\rho}$. Here, m stands for the magnetic quantum number and only m=0 is considered. This is owing to the cylindrical symmetry of the system in the case the laser field in the time-dependent interaction $H_I(t)$ is assumed to be linearly polarized along the z axis. The TDSE in (1) is solved by combining the fast Fourier transform algorithm on z-direction with the stable Cayley transform and with use of the three-point finite difference for the discretisation of the kinetic energy operator in the ρ coordinates.

The photoelectron momentum distributions stemming from the 3D calculations are shown in Fig. 1. The figures 1(a)-(d) represent the distributions for the resonant two-photon ionization (ω=10.2 eV) displayed at different peak intensities: 1 $\times 10^{14}$ W/cm^2 (Fig.1(a)), 4 $\times 10^{14}$ W/cm^2 (Fig.1(b)) and 6 $\times 10^{14}$ W/cm^2 (Fig.1(c)) where two, three and four Rabi-cycles are completed, respectively. A clear splitting of the localised momentum distribution is seen depending on the peak intensity, as discussed in the main text. The emergence of this splitting effect validates the results obtained using the 2D-model in the main text. For reference, we also show the distribution obtained in the case of one-photon ionization (ω=20.4 eV). This is shown at the peak intensity of 1.6 $\times 10^{14}$ W/cm^2, and indeed, no splitting is observed as expected and discussed in the main text.

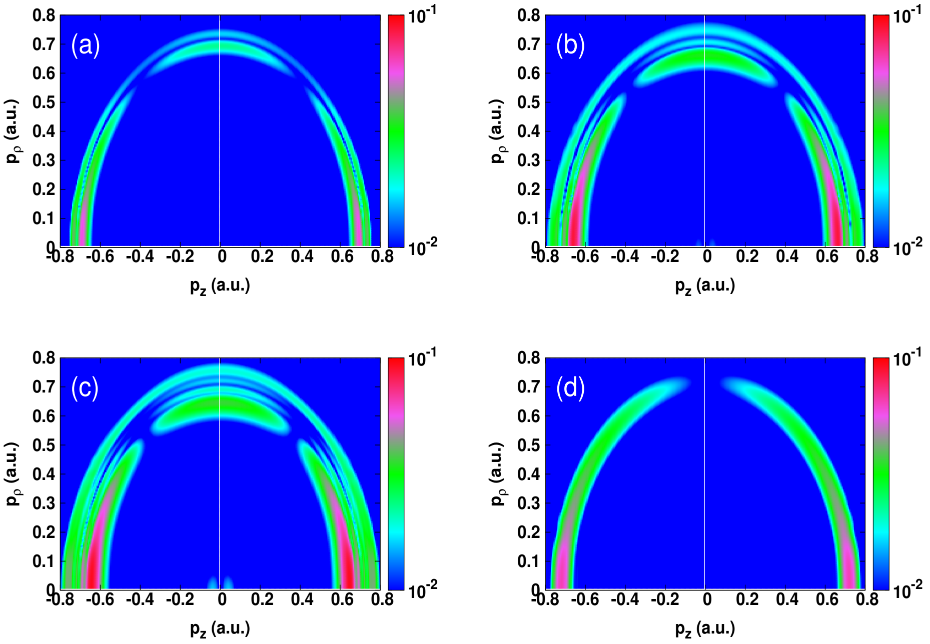

Figure 1. Photoelectron momentum distributions in the $p_\rho - p_z$ plane for the hydrogen atom. (a)-(c) Resonant two-photon ionization with the central frequency ω=10.2 eV. (d) One-photon ionization with the central frequency ω=20.4 eV. The distributions are shown for different peak intensities :(a) 1 $\times 10^{14}$ W/cm^2; (b) 4$\times 10^{14}$ W/cm^2; (c) 6 $\times 10^{14}$ W/cm^2; and (d) 1.6 $\times 10^{14}$ W/cm^2.
